# Electrically controlled spin polarized current in Dirac semimetals

**DOI:** 10.1038/s41598-021-01067-y

**Published:** 2021-11-02

**Authors:** Qianqian Lv, Pei-Hao Fu, Xiang-Long Yu, Jun-Feng Liu, Jiansheng Wu

**Affiliations:** 1 Department of Physics, Harbin Institute of Technology, Harbin, 150001 China; 2 Department of Physics, Southern University of Science and Technology, Shenzhen, 518055 China; 3 Shenzhen Institute for Quantum Science and Engineering (SIQSE), Southern University of Science and Technology, Shenzhen, 518055 China; 4 School of Physics and Materials Science, Guangzhou University, Guangzhou, 510006 China; 5 International Quantum Academy (SIQA), and Shenzhen Branch, Hefei National Laboratory, Futian District, Shenzhen, P. R. China

**Keywords:** Materials science, Mathematics and computing, Physics

## Abstract

We propose a highly tunable $100\%$ spin-polarized current generated in a spintronic device based on a Dirac semimetal (DSM) under a magnetic field, which can be achieved merely by controlling electrical parameters, *i.e.* the gate voltage, the chemical potential in the lead and the coupling strength between the leads and the DSM. These parameters are all related to the special properties of a semimetal. The spin polarized current generated by gate voltage is guaranteed by its semimetallic feature, because of which the density of state vanishes near Dirac nodes. The barrier controlled current results from the different distance of Weyl nodes generated by the Zeeman field. And the coupling strength controlled spin polarized current originates from the surface Fermi arcs. This DSM-based spintronic device is expected to be realized in $\hbox {Cd}_{3}\hbox {As}_{2}$ experimentally.

## Introduction

The generation and manipulation of spin polarized current are the key task to spintronics. One of the typical approaches to generate the spin polarization in the devices is to apply a magnetic field. However, the precise manipulation relying on a fine-tuning magnetic field remains a challenge. Recently, thanks to the discovery of topological materials in the past decades^1–3^, it is possible to realize highly tunable spintronics through electric methods.

In this work, we study the electrically controlled transport phenomena in a Weyl semimetal (WSM)^4–7^ created in a Dirac semimetal (DSM)^8–19^ by a Zeeman field. Both WSM and DSM have gained increasing interest recently, due to (i) the nature of Weyl/Dirac quasiparticles in their band structures^16–19^ and (ii) the surface Fermi arcs that connecting these nodes^13,14^. Generally, a fourfold degenerate Dirac point is composed of two double degenerate Weyl points with opposite chirality^15^. Thus a pair of Weyl nodes with two different spin subbands can be created from each Dirac node by a time-reversal breaking perturbation, such as high-frequency illumination^20–22^ or a magnetic field^23,24^. There are many transport experiments and applications on these new materials^25,26^, including superconductivity^27–30^, Aharonov-Bohm interference^31,32^ and higher-order topological states^33^. However, the proposals for DSM-based topological spintronics are still lacking.

**Figure 1:**
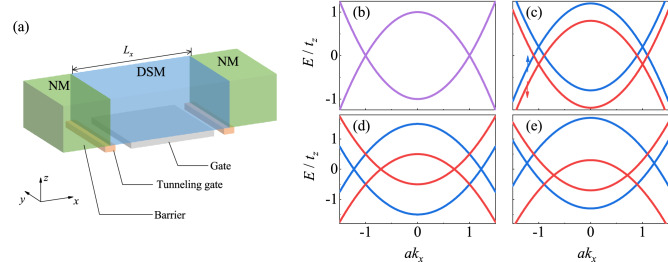
(**a**) Schematic for a normal metal (NM)/Dirac semimetal (DSM)/NM junction. The length of the junction is $L_{x}$. The polarization of the current can be controlled by the barrier, gate and tunneling gate in the junction. (**b**–**e**) Dispersions of Dirac semimetals (**b**) without and (**c**–**e**) with Zeeman field. The spin-up and spin-down subband are plotted in blue and red, respectively. The Zeeman terms $(B_{\Delta },B_{0})$ are (**b**) (0, 0), (**c**) (0, 0.2), (**d**) (0.5, 0) and (**e**) (0.5, 0.2). The dispersions are calculated using the simplified parameters shown in Table 1.

Here, inspired by two recent experiments^34,35^, we propose that a highly tunable $100\%$ spin-polarized current can be generated in a normal metal (NM)/DSM/NM junction under an external magnetic field (see Fig. 1a). The current polarization can be controlled by (i) the gate voltage applied to the central DSM, (ii) the chemical potential in the NM leads and (iii) the coupling strength between the leads and DSM. Each access involves one characteristic of DSM, including the semimetallic feature, the distances of Weyl/Dirac nodes and the surface Fermi arcs, respectively. With an external magnetic field, it is not surprising to generate a fully spin-polarized current. However, it is a challenge to control the current polarization, which usually requires an inversion of current direction^34^, the direction of magnetic field^24^. In our work, due to the separation of spin subbands caused by a Zeeman field and accompanied with the creation of Weyl nodes^23^, the current polarization is electrically manipulable, which makes a DSM as a potential topological spintronics device.

The remainders are organized as follows. The low energy model of DSM with Zeeman term and the corresponding dispersion are introduced in “Model”. The transport results and discussions in the NM/DSM/NM junction calculated from lattice Green’s function are demonstrated in “Numerical results and discussions”. The “Conclusion” is given in the final section.

**Table 1:** Value of parameters of the simplified model, $\hbox {Na}_{3}\hbox {Bi}$^15,36^ and $\hbox {Cd}_{3}\hbox {As}_{2}$^23,37^.

	$$C_{0}$$ (eV)	$$C_{x,y}$$ (eV Å$$^{2}$$)	$$C_{z}$$ (eV Å$$^{2}$$)	$$ M_{0}$$ (eV)	$$M_{x,y}$$ (eV Å$$^{2}$$)	$$M_{z}$$ (eV Å$$^{2}$$)	*v* (eV Å)	$$a_{x,y}$$ (Å)	$$a_{z}$$ (Å)	$$g_{s}$$	$$g_{p}$$
Simplified model	0	0	0	1	1	1	0.5	1	1	–	–
$\hbox {Na}_{3}\hbox {Bi}$	$-\,0.06$	8.4	8.75	$-\,0.08$	$-\,10.36$	$-\,10.64$	2.46	5.488	4.828	18.6	2
$\hbox {Cd}_{3}\hbox {As}_{2}$	$-\,0.0145$	11.5	10.59	$-\,0.0205$	$-\,13.5$	$-\,18.77$	0.889	12.64	25.43	20	20

## Model

We begin with the low-energy effective Hamiltonian of a DSM around the $\Gamma$ point^23^,

1 $$\begin{aligned} H_{D}\left( \varvec{k}\right) =\epsilon _{0}\left( k\right) \sigma _{0}\tau _{0}+M\left( k\right) \sigma _{0}\tau _{z}+v\left( k_{x}\sigma _{z}\tau _{x}-k_{y}\sigma _{0}\tau _{y}\right) \text {.} \end{aligned}$$

The Hamiltonian is written in the basis $\{|S_{J=1/2},J_{z}=1/2\rangle ,|P_{3/2},3/2\rangle ,|S_{1/2},-1/2\rangle ,|P_{3/2},-3/2\rangle \}$. And $\epsilon _{0}\left( \varvec{k}\right) =C_{0}+\sum _{i=x,y,z}C_{i}k^2_{i}$ and $M\left( \varvec{k}\right) =M_0-\sum _{i}M_{i}k^2_{i}$. This Hamiltonian is valid for $\hbox {Cd}_{3}\hbox {As}_{2}$^8^ and $\hbox {Na}_{3}\hbox {Bi}$^15^, where $C_{0,x,y,z}$, $M_{0,x,y,z}$ and *v* are model parameters fit by *ab* initio calculation as shwon in Table 1. $\varvec{\sigma }$ ( $\sigma _{0}$) and $\varvec{\tau }$ ($\tau _{0}$) are Pauli matrices (unit matrix) for the spin and orbital degree of freedom, respectively. We hereafter define the *z* axis as the spin-quantization axis. The dispersion of Eq. (1) describes spin-up (spin-down) subbands parallel (antiparallel) to the spin-quantization axis, which contain two Dirac nodes protected by crystalline symmetry and along $k_{z}$-axis at $\varvec{K}_{D}=\left( 0,0,\pm k_{D}\right)$ for $k_{D}=\sqrt{M_{0}/M_{z}}$. The Hamiltonian (1) is invariant under the time-reversal symmetry^38,39^

2 $$\begin{aligned} \hat{T}H_{D}\left( \varvec{k}\right) \hat{T}^{-1}=H_{D}\left( - \varvec{k}\right) \text {,} \end{aligned}$$

with $\hat{T}=i\sigma _{y}\tau _{0}\hat{K}$ ($\hat{K}$ is a complex conjugation) and inversion symmetry

3 $$\begin{aligned} \hat{P}H_{D}\left( \varvec{k}\right) \hat{P}^{-1}=H_{D}\left( - \varvec{k}\right) \text {,} \end{aligned}$$

with $\hat{P}=\sigma _{0}\tau _{z}$. In a DSM, each single Dirac node contains two Weyl nodes in different spin subbands because of the co-existence of time-reversal symmetry and inversion symmetry. If one or all of these symmetries are broken, the single Dirac node will be split into two Weyl nodes with opposite chirality and a DSM naturally evolves into a WSM^23^.

One of useful approaches to break the symmetries is to apply a magnetic field to the system. In a DSM, when a magnetic field is along spin-quantization axis, the Zeeman term takes the form as

4 $$\begin{aligned} H_{Z}=-\sigma _{z}\otimes \left( \begin{array}{cc} B_{s} &{} 0 \\ 0 &{} B_{p} \end{array} \right) \text {,} \end{aligned}$$

where $B_{s,p}=g_{s,p}\mu _{B}B_{z}/2$ is the effective Zeeman term causing by an orbital-dependent *g*-factor $g_{s,p}$, $\mu _{B}$ is the Bohr magneton and $B_{z}$ is the strength of the field. An orbital dependent *g*-factors is chosen, because the bands of a DSM come from different representations^23^. This is confirmed experimentally^36,37^ and theoretically^40^ and the values of the *g*-factors in $\hbox {Na}_{3}\hbox {Bi}$ and $\hbox {Cd}_{3}\hbox {As}_{2}$ are shown in Table 1. Here, in order to clarify the effect of magnetic field, Eq. (4) can be rewritten as

5 $$\begin{aligned} H_{Z}=B_{0}\sigma _{z}\tau _{0}+B_{\Delta }\sigma _{z}\tau _{z}\text {,} \end{aligned}$$

with $B_{0}=-\left( B_{s}+B_{p}\right) /2$ and $B_{\Delta }=-\left( B_{s}-B_{p}\right) /2$. It is noted that the first term in Eq. (5) breaks both $\hat{T}$ and $\hat{P}$ while the second term only breaks $\hat{T }$. These effects are reflected in energy spectrum of the Hamiltonian

6 $$\begin{aligned} H_{W}=H_{D}+H_{Z}\text {,} \end{aligned}$$

whose dispersion is

7 $$\begin{aligned} E_{\pm }^{\sigma }=\epsilon _{0}\left( k\right) +\sigma B_{0}\pm \sqrt{\left[ M\left( k\right) +\sigma B_{\Delta }\right] ^{2}+v^{2}\left( k_{x}^{2}+k_{y}^{2}\right) }\text {,} \end{aligned}$$

where $\sigma =+1$ ($-1$) denotes spin-up (spin-down) subbands.

To exhibit our results more clearly, the simplified model parameters depicted in Table 1 are used, while the results with model parameters of $\hbox {Cd}_{3}\hbox {As}_{2}$ and $\hbox {Na}_{3}\hbox {Bi}$ are given in the “Discussion” section. In the simplified model parameters, in order to investigate the effect of various $\left( B_{0},B_{\Delta }\right)$, no restriction is made on the orbital-dependent *g*-factors. Figure 1b–e depict the dispersions with different $\left( B_{0},B_{\Delta }\right)$. In the absent of magnetic field, the system is a DSM with two Dirac nodes at $\varvec{K}_{D}=\left( 0,0,\pm k_{D}\right)$ as shown in Fig. b. When applied magnetic field, each Dirac node splits into two Weyl nodes at $\varvec{K}_{W}^{\sigma }=\left( 0,0,\pm k_{W}^{\sigma }\right)$, $k_{W}^{\sigma }=\sqrt{\left( \sigma B_{\Delta }+M_{0}\right) /M_{1}}$, with opposite chirality $\chi _{\pm }^{\sigma }=\pm \sigma sign\left( B_{\Delta }\right)$. There are three cases for splittings. (i) When the effects of magnetic field on two orbits are identical ($B_{\Delta }=0$), two spin subbands are shifted in an opposite direction on energy scale resulting two pairs of Weyl nodes with different energy (see Fig. 1c). (ii) For another case where $B_{0}=0$ (see Fig. 1d), each Dirac node splits into two Weyl nodes along $k_{z}$-axes. (iii) Finally, combining two cases above, a general result is obtain with finite $B_{0}$ and $B_{\Delta }$ as shown in Fig. 1e, where each Dirac node is split into two Weyl nodes in both momentum and energy scales.

## Numerical results and discussions

The creation of Weyl fermions in DSM with a external magnetic field or a magnetic doped DSM has been demonstrated explicitly^23^, which is characterized by some transport signatures such as negative magnetoconductance and three-dimensional quantum Hall effect^40^. Here, in addition to the signature of Weyl nodes creation, we focus on the electrically controlled spin polarized current. The current is generated in a junction of a magnetic-DSM sandwiched by two NM leads (see Fig. 1a), which is described by a Hamiltonian

8 $$\begin{aligned} H=H_{D}^{\prime }+H_{NM}+H_{C} \text {,} \end{aligned}$$

where $H_{D}^{\prime }$ and $H_{NM}$ describe Hamiltonian of the DSM and NM and $H_{C}$ is the coupling at the $x=0$ and $L_{x}$ interfaces. Discretizing Eq. (6) along the *x* direction, one obtains

9 $$\begin{aligned} H_{D}^{\prime }=\sum _{k_{y},k_{z},x}\left( H_{0}+H_{Z}\right) C_{k_{y},k_{z},x}^{\dagger }C_{k_{y},k_{z},x}+H_{x}C_{k_{y},k_{z},x}^{\dagger }C_{k_{y},k_{z},x+1}+H.c. \text {,} \end{aligned}$$

where

10 $$\begin{aligned} H_{0}= & {} \left( \mu _{D}+C_{0}+\sum _{i}C_{i}a_{i}^{-2}\right) \sigma _{0}\tau _{0}+\left( M_{0}-\sum _{i}M_{i}a_{i}^{-2}\right) \sigma _{0}\tau _{z} \nonumber \\&-2\left( C_{z}\sigma _{0}\tau _{0}-M_{z}\sigma _{0}\tau _{z}\right) a_{z}^{-2}\cos k_{z}a_{z}-2\left( C_{y}\sigma _{0}\tau _{0}-M_{y}\sigma _{0}\tau _{z}\right) a_{y}^{-2}\cos k_{y}a_{y}-iva_{y}^{-1}\sigma _{0}\tau _{y}\sin k_{y}a_{y}\text {,} \end{aligned}$$

and

$$\begin{aligned} H_{x}=C_{x}\sigma _{0}\tau _{0}-M_{x}\sigma _{0}\tau _{z}-iv\left( 2a_{x}\right) ^{-1}\sigma _{z}\tau _{x}\text {.} \end{aligned}$$

Here, $\mu _{D}$ is the chemical potential in DSM, which is controlled by the gate voltage, $a_{i}$ is the lattice constant in *i*-direction and *H*.*c*. denotes the Hermitian conjugate. $C_{k_{y},k_{z},x}$ ($C_{k_{y},k_{z},x}^{ \dagger }$) is the annihilation (creation) operator of electrons at site *x* with momentum $\left( k_{y},k_{z}\right)$.

**Figure 2:**
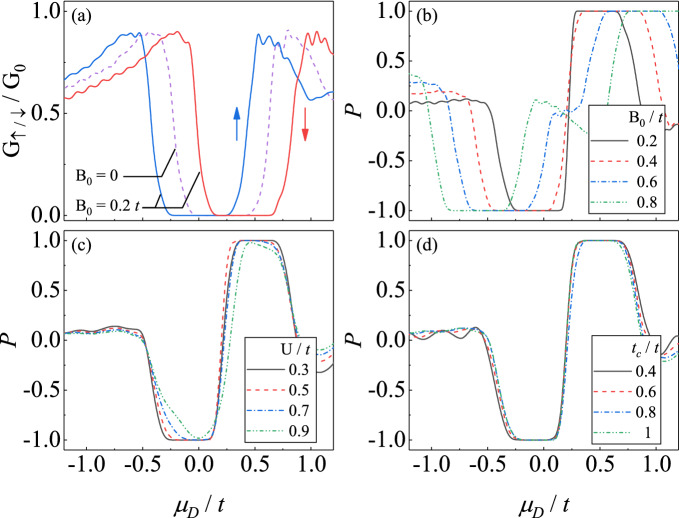
(**a**) The spin-dependent conductance with and without $B_0$. (**b**–**d**) The spin polarization *P* via the gate voltage $\mu _D$ with various (**b**) Zeeman terms $B_0$, (**c**) barriers *U* and (**d**) coupling strength $t_c$. The length of the junction is $L_x=50a$, the barrier is $U=0.3t$, the coupling strength is $t/tc=1$ and the energy of incident electron is $E=0.2t$. Other parameters are the same as those in Fig. 1c.

Similarly, the Hamiltonian of NM leads is

11 $$\begin{aligned} H_{NM}=\sum _{k_{y},k_{z},x}U\left( k_{y},k_{z}\right) \sigma _{0}\tau _{0}C_{k_{y},k_{z},x}^{\dagger }C_{k_{y},k_{z},x}-t\sigma _{0}\tau _{0}C_{k_{y},k_{z},x}^{\dagger }C_{k_{y},k_{z},x+1}+H.c\text {,} \end{aligned}$$

where, $U\left( k_{y},k_{z}\right) =\left( 6t-U-2t\cos k_{z}a-2t\cos k_{y}a\right)$ and $t=|M_{z}|a_{z}^{-2}$ is the hopping energy in the NM region, *U* is the chemical potential controlled by barriers. The Hamiltonian describing the coupling between NM leads and the DSM is

12 $$\begin{aligned} H_{C}=-t_{c}\sum _{k_{y},k_{z},x}C_{k_{y},k_{z},x}^{\dagger }C_{k_{y},k_{z},x+1}+H.c.\text {,} \end{aligned}$$

where the coupling strength $t_{c}$ can be controlled by the tunnelling barriers. The conductance of the junction is expressed as a quantum mechanical scattering probabilities and can be simply related to the total transmission probability $T\left( k_{y},k_{z},E\right)$, as

13 $$\begin{aligned} G=\frac{e^{2}}{h}\sum _{k_{y},k_{z}}T\left( k_{y},k_{z},E\right) \end{aligned}$$

where $T\left( k_{y},k_{z},E\right) =Tr\left[ \Gamma _{L}G_{LR}^{r}\Gamma _{R}G_{LR}^{a}\right]$. $\Gamma _{L/R}=i\left( \Sigma _{L/R}^{r}-\Sigma _{L/R}^{a}\right)$ is the linewidth function with $\Sigma _{L/R}^{r}$ the self-energy due to the coupling between the left/right NM lead and DSM region. And $G_{LR}^{r}=\left( E-H_{0}-\Sigma _{L}^{r}-\Sigma _{R}^{r}\right) ^{-1}$ is the retarded Green’s function, which can be obtain by means of the lattice Green’s function technique^41–43^. Since the conductance of the junction is contributed by electrons with different spin individually, the conductance can be rewritten as

14 $$\begin{aligned} G=G_{\uparrow }+G_{\downarrow }\text {,} \end{aligned}$$

with $G_{\uparrow /\downarrow }=\frac{e^{2}}{h}\sum _{k_{y},k_{z}}T_{\uparrow /\downarrow }\left( k_{y},k_{z},E\right)$, where $T_{\uparrow /\downarrow }\left( k_{y},k_{z},E\right)$ is the block matrix component in the transmission matrix $T\left( k_{y},k_{z},E\right)$. The spin polarization of the current can be defined as

15 $$\begin{aligned} P=\frac{G_{\uparrow }-G_{\downarrow }}{G}\text {.} \end{aligned}$$

In the following the simplified model parameters depicted in Table 1 are used, while the results with model parameters of $\hbox {Cd}_{3}\hbox {As}_{2}$ and $\hbox {Na}_{3}\hbox {Bi}$ are given in the end of this section.

### Gate-controlled spin-polarized current

In the absent of a magnetic field, the system is invariant under both time-reversal symmetry and inversion symmetry, thus the conductance contributed by electrons with spin-up and spin-down are identical and not spin-polarized current is created. This feature is shown in the behavior of conductance via the gate voltage in DSM region, $\mu _{D}$, in the dashed line in Fig. 2a where $G_{\uparrow }=G_{\downarrow }$ for all value of $\mu _{D}$. It should be noted that there is region of $\mu _{D}$ where $G_{\uparrow /\downarrow }=0$, which originates from the vanishing density of state (DOS) near the Dirac nodes which is the characteristic of semimetals.

In the present of a magnetic field, the spin degeneracy is shifted. We first focus on the case with $\left( B_{0},B_{\Delta }\right) =\left( 0.2t,0\right)$ exhibited in Fig. 2a, where the conductances contributed by electrons with spin-up and spin-down spilt in an opposite direction in $\mu _{D}$. As a result, there are some regions in $\mu _{D}$ where the conductance of spin-up (spin-down) subband is finite while the one of spin-down (spin-up) subband vanishes, resulting to a spin-polarized current with $P=+1$ ($-1$). In this case, the spin-polarization *P* via $\mu _{D}$ is exhibited in the black line in Fig. 2b. It is obvious that there are two platforms where $P=+1$ or $-1$ corresponding to conductance solely contributed by spin-up or spin-down electrons, respectively. In the region between two platforms, there is a high sensitivity of spin-polarization *P* to the gate voltage $\mu _{D}$, which means a slightly change in $\mu _{D}$ leads to a significant change in *P*. On the contrary, in the region out of two platforms the magnitudes of *P* rapidly reduce to zero and become insensitive to $\mu _{D}$.

These highly tunable spin-polarization is generated because both $\hat{T}$ and $\hat{P}$ are broken by $B_{0}$, which cause each Dirac node splits into two Weyl nodes in energy scale. When calculating the conductance in Fig. 2, we keep the Fermi level in the system unchange, but the energy of Weyl nodes is controlled by the gate voltage $\mu _{D}$. When one pair of Weyl nodes are tuned to near the Fermi level, because of the semimetallic feature, the DOS of the corresponding spin subband vanishes while the one of the spin subband is finite. This is the reason why the spin-polarized current occurs and can be controlled by the gate voltage $\mu _{D}$. When $B_{0}$ increases, the energy difference between Weyl nodes in two spin subbands grows leading to the distance between two spin-polarized conductance platform increases as shown in Fig. 2b. In addition, since the spin-polarized current in this case is created by breaking $\hat{T}$ and $\hat{P}$ through the magnetic field, it is robust against the parameters in the NMs (see Fig. 2c) and the interface between leads and DSM (see Fig. 2d).

**Figure 3:**
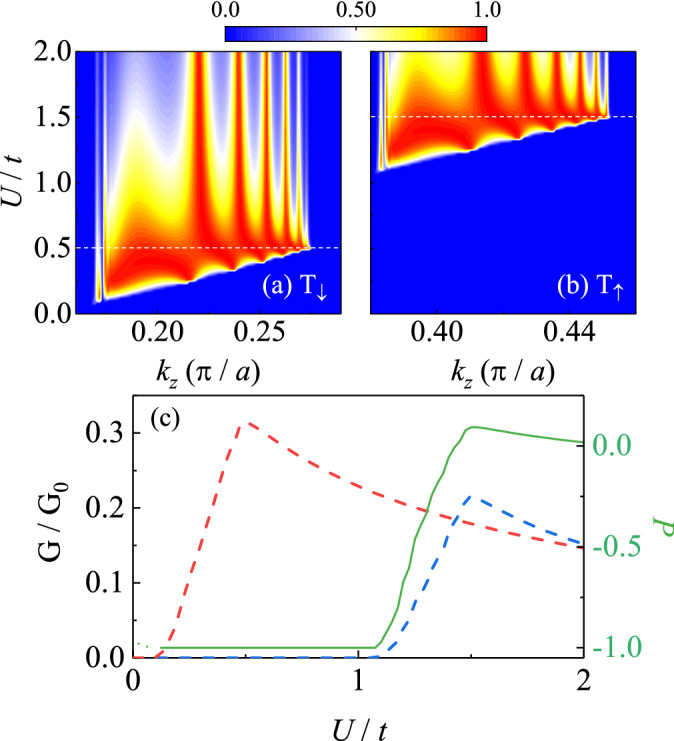
The dependence of Transmission coefficients on both *U* and $k_z$ of the (**a**) spin-down and (**b**) spin-up subband near the corresponding Weyl nodes. (**c**) The spin-dependent conductance (red and blue dashed lines) and the spin polarization (green line) via the barrier *U*. We choose $B_\Delta = 0.5k_{cz}^2$ and $B_0=0$. Other parameters are the same as those in Fig. 2.

### Barrier-controlled spin-polarized current

**Figure 4:**
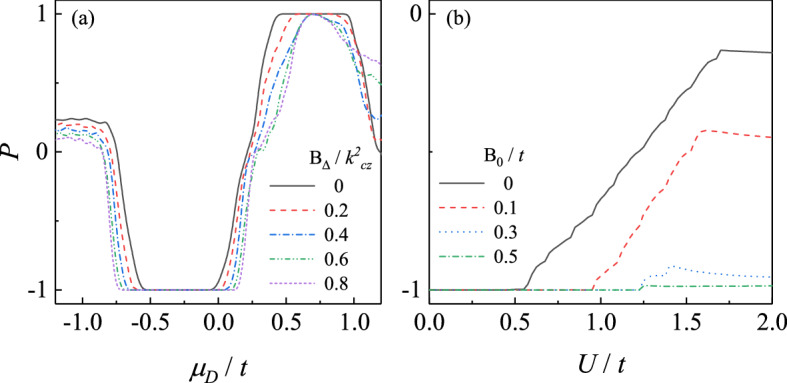
The dependence of spin polarization on (**a**) gate voltage and (**b**) barrier with different Zeeman terms. The parameters in (**a**) are the same with those in Fig. 2 with $B_0=0.2t$ while parameters in (**b**) are the same with those in Fig. 3 with $\mu _D=-\,0.2t$.

In the situation with $\left( B_{0},B_{\Delta }\right) =\left( 0,0.5k_{D}^{2}\right)$, contrary to the above situation, the present of $B_{\Delta }$ leads to four Weyl nodes in momentum space at the same energy as shown in Fig. 1c. The system now is in WSM phase with both two spin subband holds a pair of Weyl nodes with opposite chirality when the Fermi level is near the nodes. Without splitting in energy scale, it is difficult to generate spin-polarized current by means of the gate voltage $\mu _{D}$. However, since the different locations in momentum space of two pairs of Weyl nodes, spin-polarized current can be obtained by controlling *U*, the chemical potential in NM region.

The dependence transmission coefficients $T_{\downarrow ,\uparrow }$ on both *U* and $k_{z}$ are exhibited in Fig. 3a,b. Both of $T_{\downarrow }$ and $T_{\uparrow }$ are nonzero only in a small region around the Weyl nodes in the corresponding subband. However, since the distance of nodes in the spin-up subband is larger than those in the spin-down subband, the spin-up transmission coefficients $T_{\uparrow }$ is lagged behind the spin-down one, leaving a wide region of *U* where $T_{\uparrow }=0$ but $T_{\downarrow }\not =0$. This gives rise to the spin polarized current controlled by *U* as shown in Fig. 3c, where the conductance contributed by spin-up and spin-down electrons are shown separately. For a small *U*, the spin polarization is not well defined, since both the conductances are nearly zero. When *U* grows, the spin-down conductance begins to increase in advance to the spin-up one, leading to $P=-1$. As *U* is large enough that the spin-up electron are involved in the transmission process, the polarization vanishes. Our numerical results also reveals that the spin-polarization current is robust against the interface coupling between leads and DSM.

Combining the results in the last subsection, in the general case where both $B_{0}$ and $B_{\Delta }$ are finite, highly tunable spin-polarized current can be generated by controlling the gate voltage $\mu _{D}$ and the barrier *U* in the junction, which are shown in Fig. 4. It should be noted that the polarization is still sensitive to the gate voltage $\mu _{D}$ and can be switched between $P=-1$ and $+1$ in a wide region of $B_{\Delta }$.

### The role of Fermi arcs

**Figure 5:**
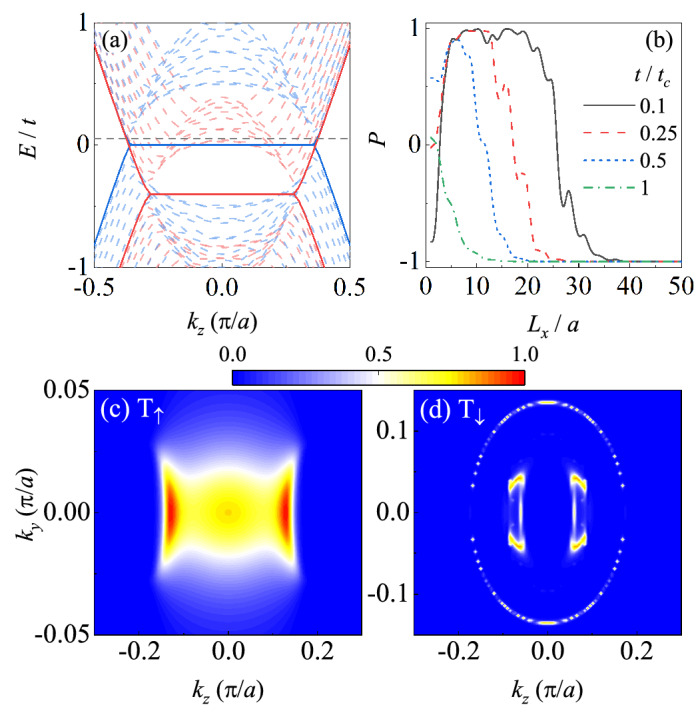
(**a**) The spectrum of DSM with Zeeman terms $B_\Delta =0.2k_{cz}^2$ and $B_0=0.2t$ in open boundary condition along *x*-direction with $L_x=100$ , $k_y=0$ and $\mu _D=-0.2t$. (**b**) The spin polarization via the length of the junction with various coupling strengths. (**c**,**d**) The momentum-resolved transmission coefficient $T_{\uparrow }$ ($T_{\downarrow }$) in a junction with length $L_x = 10a$ (30*a*) with the coupling strength $t_c=0.25t$. We choose $U=0.3t$ and other parameters are the same as those in Fig. 4b.

The results so far focus on the properties of bulk states in a magnetic DSM. However, the spin-polarized Fermi arc surface state is another characteristic of DSM^13,14^, whose effects on the conductance is missed above. In Fig. 5a, the spectrum of magnetic DSM involving the surface states are shown, which is calculated from a Eq. (9) with periodic boundary condition in both *z*- and *y*-direction and open boundary condition in the *x*-direction. The flat band in Fig. 5a denotes the surface Fermi arcs connecting bulk Weyl nodes. In our proposal, because of the broken translational symmetry along *x*-direction when constructing the junction, Fermi arcs surface state is localized at the *y*-*z* plane with a group velocity along *y*-direction and dispersionless along *z*-direction [the completely flat band in Fig. 5a As a result, the surface state can contribute to the current through evanescent modes at two lead-DSM interfaces.

The result shown in Fig. 4b is in a long junction limit, where the length exceeds the penetration lengths of the surface state and the bulk states becomes dominated in the current. In this limit, the spin-down electrons dominate the conductances leading to $P=-1$ when the length of the junction $L_{x}/a$ is large for various coupling strength $t_{c}$.

However, it is interesting that the polarization of the current inverses in a short junction with a weak coupling. Now, instead of the propagating mode and the bulk modes, the surface state can contribute to the current through evanescent modes at two lead-DSM interfaces. When length of the junction is comparable to the penetration lengths of the surface state, electrons in the left lead can transmits to the right lead through the overlap between the evanescent surface modes at $x=0$ and $x=L_x$ surface, which is the current contributed by the Fermi arc surface state. In the short junction limit, the contribution of the bulk state is suppressed, because of the bulk gap is induced by the finite size effect. Besides, the reducing coupling produces a potential barrier at the interface between DSM and lead, increasing the tunneling conductance mediated by the Fermi arc surface states localized at two interfaces and closed to the Fermi level. As exhibited in Fig. 5a, the spin-up Fermi arc is near to the Fermi level (the gray dashed line) generating a spin-up electrons dominated current with $P\sim +1$ in the short junction and weak coupling limit shown in Fig. 5b. This can be confirmed by the momentum-resolve spin-up transmission coefficient $T_{\uparrow }$ in Fig. 5c in the short junction limit, while the $T_{\downarrow }$ nearly vanishes. In Fig. 5c, $T_{\downarrow }$ is dominated by the modes between Weyl nodes, which is the contribution of the surface states. On the contrary, in a long junction whose length exceeds the penetration lengths of the surface state, the bulk states becomes dominated in the current. This is justified by the momentum-resolve spin-down transmission coefficient $T_{\downarrow }$ in Fig. 5d, which is mainly contributed by the modes around the Weyl cones.

### Discussions on experimental realization

**Figure 6:**
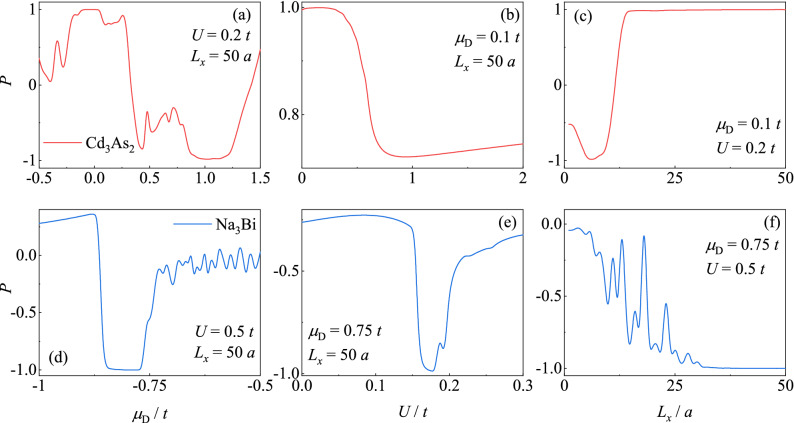
The current polarization tuned by (**a**,**d**) voltage gate, (**b**,**e**) barrier and (**c**,**f**) the length of the junction made by $\hbox {Cd}_{3}\hbox {As}_{2}$ (top panel) and $\hbox {Na}_{3}\hbox {Bi}$ (bottom panel). The model parameters are shown in Table 1. The Fermi level is $E=0.2t$ and the magnetic field is $B_z = 5$ T. Other parameters are shown in the figures.

The above results are obtained from the simplified model parameters. In this subsection, the realization of our proposal is discussed using the the first-principle-fit parameters in Table 1, which may serve as a reference for future experiments. The effect of Landau level is beyond our current discussions, which is another method to generate spin-polarized current in DSM controlled by a lateral gate^24^. Here, this orbital effect can be ignored because the required field strength is of the order of $B_z\sim 5$ T, resulting to $B_za_z^2\ll h/2e$.

Figure 6 shows the results involving two well-accepted DSM candidate materials, $\hbox {Cd}_{3}\hbox {As}_{2}$^9–13^ and $\hbox {Na}_{3}\hbox {Bi}$^14,15^. In $\hbox {Cd}_{3}\hbox {As}_{2}$ (see the top panel of Fig. 6), since $g_s=g_p$, the current is fully spin-polarized and the polarization can is highly tunable by the gate voltage. Although the polarization is insensitive to the chemical potential because of the identical *g*-factor of two orbits, a fully spin-polarized current can be generated with suitable parameters. Besides, the Fermi-arc-contributed current polarization is also expected. On the contrary, in $\hbox {Na}_{3}\hbox {Bi}$, although it is possible to generate the fully spin-polarized current, the polarization of the current is difficult to manipulate and the current contributed by the surface state is ambiguous. In a word, although affected by the $C_{0,x,y,z}$ parameters sightly, compared with $\hbox {Na}_{3}\hbox {Bi}$, our proposal is expected to be realized in $\hbox {Cd}_{3}\hbox {As}_{2}$.

## Conclusion

In this work, it is found that a highly tunable $100\%$ spin-polarized current can be generated in an NM/DSM/NM junction, and it can be achieved only by controlled one of the three electric parameters, *i.e.* the gate voltage applied to the central DSM, the barrier in the NM leads, and the coupling strength between the leads and DSM. These three methods reveal three aspects of DSM. The spin polarized current generated by gate voltage is guaranteed by its semimetallic feature, because of which the DOS vanishes near Dirac nodes. The barrier controlled current is generated resulting from the different distance of Weyl nodes in the corresponding spin subbands. While all these two feature are caused by the bulk properties of DSM, the coupling strength controlled spin polarized current originates from the surface Fermi arcs, which is another characteristic of DSMs. All these three features make a great potential to realize DSM-based spintronics devices merely controlled by electric methods and we expect that our proposals can be realized in $\hbox {Cd}_{3}\hbox {As}_{2}$.
